# Resilience assessment tool for port planning

**DOI:** 10.1177/2399808321997824

**Published:** 2021-03-08

**Authors:** Manhar Dhanak, Scott Parr, Evangelos I Kaisar, Panagiota Goulianou, Hannah Russell, Fanny Kristiansson

**Affiliations:** 1782 Florida Atlantic University, USA; Embry–Riddle Aeronautical University, USA; Freight Mobility Research Institute, 1782 Florida Atlantic University, USA; 1782 Florida Atlantic University, USA; Embry–Riddle Aeronautical University, USA

**Keywords:** Port resilience, integrated hybrid port modeling, resilience quantification

## Abstract

US ports and container/intermodal terminals are critical links in the marine transportation system. Disruption at a port can have a crippling economic effect in the coastal zone as well as the rest of the nation. Port stakeholders have a vested interest in the long-term function and viability of ports, but no standardized measures for performance or resilience exist for ports. The goal of this research is to demonstrate the utility of a predictive port resilience assessment tool. The developed tool encompasses a microscopic traffic simulation model (VISSIM) based hybrid multimodal analyzes of port operations and provides a quantifiable assessment of resilience. The application of this tool is shown on six ports in the Southeast US. The waterside port simulation models were developed using vessel automatic identification system data and programed within a VISSIM simulation of landside operations. This hybrid modeling approach was used to visualize vessels and allow them to interact in both time and space with each other and landside infrastructure. Local and regional resilience was quantified through the analysis of time-dependent resilience plots and used as a performance measure in this study. The utility of the predictive port resilience assessment was demonstrated in response to Hurricane Matthew (2016). However, the novel procedure described herein can be applied to any port hazard. This research grows the understanding of the regional consequences of hurricane events and enhances the knowledge in the development of a stakeholder-focused tool to assess resilience.

## Introduction

The National Academies define resilience as “the ability to prepare and plan for, absorb, recover from, or more successfully adapt to actual or potential adverse events” (National Research Council (NRC), 2012). Resilient infrastructure provides the means to deliver the essential goods and supplies needed to safely and quickly recover from a storm, attack, or other major disruption. Resilient ports help to create a shorter disruptive period and a faster time to recovery. As such, there is a need to better understand the relationship between port operations, disruptions, and resilience. Quantitative methods and tools, stemming from engineering science and vulnerability studies, provide quick assessments of “resilience” at broad spatial scales, but do not dip below the surface into local scale, place-based, community resilience. Qualitative methods, on the other hand, help answer research questions that cannot be addressed with numerical data and dive into questions of attitude, perception, and social interaction.

An approach to measuring resilience must be adaptable to the specific needs of the community using it, which quickly renders a national-scale resilience metric nearly impossible. Driven by global economic forces, ports have unique needs that should inform indicators to assess resilience over time. The goal of this research was to demonstrate the utility of a port resilience assessment tool. The developed tool involved a VISSIM-based hybrid multimodal simulation that analyzed port operations and provided a quantifiable assessment of resilience. VISSIM is a state-of-the-art microscopic transportation simulation model commercially developed by PT VISION. The application of this tool was shown on six ports in the Southeast US. The waterside port simulation models were developed using vessel automatic identification system (AIS) data and programed within a VISSIM simulation of landside operations. This hybrid modeling approach was used to visualize vessels and allows them to interact in both time and space with each other and landside port infrastructure. VISSIM also provided a means of analyzing vessel queuing and data extraction. Resilience was quantified through the analysis of time-dependent resilience plots and used as a performance measure in this study. The utility of the predictive port resilience assessment was demonstrated in response to Hurricane Matthew. However, the novel procedure described herein can be applied to any local or regional port hazard.

This research sought to grow the understanding of port resilience through to development of a stakeholder-focused tool to improve port resilience. This research used knowledge, innovation, and education, as well as modeling and simulation, to assess port resilience. Given the nature of resilience as a dynamic process, this study considers strategies for managing identified risk. The approach allowed for an assessment of port resilience and improvement in understanding of the consequences of hurricane events at the ports and intermodal facilities within a region, in support of seeking improvements in the regional preparedness of interconnected port systems. The application of the predictive port resilience assessment tool can lead to more informed decision making for emergency managers and port officials. The tool objectively evaluated the resilience of local and regional ports in response to policy, protective actions, decisions, and infrastructure improvements. The dynamic simulation architecture allows for the evaluation of various hazards, timescales, and regions. Port planners may also find this tool useful to demonstrate the benefits of investment in resilience, using quantifiable metrics.

## Literature review

The Center for Transportation & Logistics at the Massachusetts Institute of Technology (MIT) conducted a multiyear Port Resilience project (Rice et al., 2013). The goal of the study was to estimate the capacity required to absorb various failures of United States ports. The project included a port capacity analysis, port failure mode analysis, and a detailed port resilience survey. In addition, they developed a platform called MIT Port Mapper, which was designed to identify U.S. ports that can potentially absorb cargo in the event of a port disruption. The user chooses the desired state and port to examine. In addition, the platform used U.S. Army Corps of Engineers (ACE) data for gathering information on the type of materials handled in each port (e.g., radioactive, containers, etc.).

The Americas Relief Team (2013), in collaboration with FedEx, conducted a project entitled “Port Resiliency Program.” Its objective was the preparation of airports and seaports in the Caribbean and Latin America to be more resilient in the face of natural disasters by applying lessons learned in Hurricane Katrina (2005) and the 2010 Haiti earthquake. Their approach for achieving their goal comprised of three main steps: Initial self-assessment by the airport or seaport; planning of a workshop in Miami to identify gaps and training needs in sea and airport operations; and a site visit to present training and a tabletop exercise to assess the preparedness of the airport or seaport.

In addition, Mansouri et al. (2011) conducted a research project entitled “A Policy Making Framework for Resilient Port Infrastructure Systems.” This work developed a Risk Management-based Decision Analysis framework with the goal of forming a systematic process for making strategic and investment decisions in case of disruptions. The disruption cases considered ranged from natural disasters to organizational, technological, and human factors. Their approach can help to identify common elements of uncertainty in port systems, evaluate the costs incurred with various potential failures and with investing in resilience strategies. In their paper, Nair et al. (2010) discussed ports and intermodal freight systems, highlighting the dangers that hinder cargo transportation and the infrastructure’s vulnerability to disasters. They quantified resilience as the post disruption fraction of demand that can be satisfied while using specific available resources and managing to maintain a prescribed level of service. Additionally, they employed their concept on a system level and proposed a generic framework for its application in intermodal facilities. In another paper, resilience was measured and maximized for freight transportation networks. The model, apart from measuring resilience levels of a freight network, included the optimal setting of actions and the allocation of budget between preparedness and recovery activities under level-of-service constraints (Miller-Hooks et al., 2012).

Moreover, a paper dealing with the quantification of resilience by Pant et al. (2014) applied stochastic measures of resilience to container terminals. The authors modeled the system resilience as a function of both vulnerability and recoverability, while also incorporating aspects of stochasticity and uncertainty in terms such as time to total system restoration and time to full system service resilience. The resilience decision-making framework that was created included commodity flows at a port, full or partial terminal closures due to disruptive events and restoration activities, and it was applied in a case study at the port of Catoosa in Oklahoma.

Furthermore, the Gulf of Mexico Alliance conducted a study to develop a ports resilience index and identified three case studies in the Gulf of Mexico (Morris, 2016). The objective of the project was to produce a simple and easily implementable regional tool that port and marine transportation authorities could use to evaluate and assess their level of resilience, as well as predict their ability to achieve an acceptable level of service during and after major weather events. The Ports Resilience Index was constructed using the Delphi Method, commonly used for quantifying variables of uncertainty and reaching a statistical consensus. The case studies considered were the Port of Corpus Christi in Texas, the Port of Pascagoula in Mississippi, and the Port of Lake Charles in Louisiana.

Port modeling takes on multiple aspects to obtain the most realistic model of the area. Since ports are a transfer point for cargo, they have unique characteristics and can be affected by both landside and seaside disruptions. Work on this topic by John et al. (2015) suggested that “when critical maritime systems do not have the robustness to recover in the face of disruption, they present themselves as attractive targets to terrorism-related attacks” and “disruptions at any point within their operation could potentially result in catastrophic and disastrous consequences.” These insights were reiterated by Alyami et al. (2019) stating, “safe and reliable operations are of great significance for the protection of human life and health, the environment, and the economy” and “early detection of hazards was crucial in avoiding performance degradation and damage to human life and property.”

Ports are more than just a single location, but a critical link in a supply chain that can span the globe. Port modelers must acknowledge the fact that in a globalizing economy, the competition among ports includes the supply chains they could potentially serve (Magala and Sammons, 2008). When modeling a port, the entire system must be examined. To this end, Al-Deek (2001) developed a trip generation and modal split model as part of a four-step transportation planning process to estimate the number of truck and rail trips generated by the vessel cargo. Traffic movements within a port can be as high as 2000 per day and the number is expected to continue to increase (Debnath et al., 2011). AIS can be used to help model ports by allowing for classifying vessel patterns (Chen et al., 2018).

## Methodology

In the methodology, we first define an impartial, quantitative approach for evaluating resilience that would enable a robust performance metric. We then discuss developing, calibrating, and validating the hybrid multimodal simulation model of six ports located in the Southeastern United States impacted by Hurricane Matthew (Port of Miami, Port Everglades, Port Canaveral, Port of Jacksonville, Port of Savannah, and Port of Charleston). Finally, we discuss the development and programing of three evaluation scenarios: *Baseline*, *Hurricane Matthew*, and *Capacity Enhanced*.

### Resilience definition and assessment

**Figure 1.**
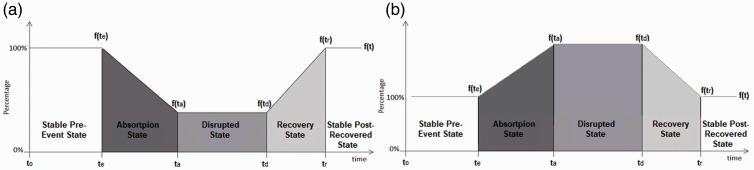
(a) Time-dependent resilience plot increasing service system. (b) Time-dependent resilience plot decreasing service system.

The National Academies’ definition of resilience calls for a means of measuring the system’s ability to absorb, adapt, and recover (NRC, 2012). This provides insight into how resilience can be quantified. Figure 1(a) shows a generic time-dependent resilience plot for an increasing service system (the dependent variable increases as service increases) undergoing a disruptive event. Figure 1(b) provides an example of a decreasing service system (the dependent variable decreases as service increases) experiencing a disruption. Henry and Ramirez-Marquez (2012) first proposed using time-dependent service functions as means of measuring system resilience. The work presented in this paper seeks to build upon the existing literature and provides a novel formulation for quantifying resilience. Let function f(t) represent a direct measure of system performance at any time *t*. System *S* will undergo five distinctive states. Prior to event *e* ( $t<t_{e}$ ), the system is operating in the Stable Pre-Event state. After event *e*, the output decreases as the system absorbs the impact of the disruption. During this period, when performance is decreasing, the system is in the Absorption state, $t_{e}\leq t<t_{a}$ . Eventually, the system will stabilize as the effect of the disruption reaches its maximum impact on performance. While system performance is no longer decreasing, the system operates in the Disrupted state as output is still reduced from the pre-event conditions $\;f\left(t_{a}\right)≅f\left(t_{a+1}\right)\neq f(t_{e-1})$ . The system will remain in the Disrupted state until a recovery action is taken at $t=t_{d}$ . The system begins to recover as the performance increases during the Recovery state, $f(t_{D+1})>f(t_{D})$ . This recovery continues until the system reaches a Stable Post-Recovered state at $t=t_{R}$ .

The system functionality between $t_{e}\;$ and $t_{a}\;$ can be used as a direct measure of absorption. In particular, the angle created within the plot is an intuitive measure of the system’s ability to absorb. This value can also be normalized between zero and one, by the inverse tangent function. Equation (1) represents the system’s ability to absorb the impact of the event. If the Absorption state is 1.0, the disruption has no effect on the system. However, a sharp, negative slope indicates poor absorption and results in a value closer to zero.

(1) $$R_{A}=1-\frac{2}{\pi }\left|{\text{tan}}^{-1}\left(\frac{f\left(t_{a}\right)-f(t_{e})}{t_{a}-t_{e}}\right)\right|$$

Similarly, the system’s ability to recover can also be measured by the angle created in the resilience plot within the Recovery state, $t_{D}<t<t_{R}$ . Equation (2) quantifies the system’s recovery after a recovery action has been taken.

(2) $$R_{R}=\frac{2}{\pi }\left|\tan^{-1}\left(\frac{f\left(t_{r}\right)-f(t_{d})}{t_{r}-t_{e}}\right)\right|$$

The functionality during the Disrupted state represents the system’s ability (or lack thereof) to adapt to the adverse conditions and overcome the disruption. While system performance is no longer decreasing, the inability to “bounce back” is measured in the Disrupted state. Equation (3) provides a measure, between zero and one, for the system’s ability to quickly adapt to the new conditions, which exist after the disruption.

(3) $$R_{d}=1-\frac{t_{e}-t_{a}}{t_{r}-t_{e}}$$

Resilience is a measure of the system’s absorption (equation (1)), recovery (equation (2)), and adaptation (equation (3)), and then a quantifiable measure of resilience is given as equation (4).

(4) $$R=R_{A}*R_{D}*R_{R}\;$$

This formulation of resilience suggests that if the system is unable to absorb or adapt or recover, it is, effectively, not resilient, i.e., R=0 .

### Hybrid multimodal simulation model development

Broadly, two simulation models were developed for each port: landside and waterside. These two models were integrated into a single, unified hybrid multimodal model to simulate a 30-day period of port operations. The micro-simulation tool VISSIM was selected as the simulation platform for this study. VISSIM provides an opportunity to integrate the models and visualize port operations. Along with the visual representation, this tool provided a vast array of performance measures that were used to describe, in detail, how the ports were affected by emergency events. The landside models used publicly available data provided by Florida, Georgia, and South Carolina state-run departments of transportation for their development and calibration while the waterside models used purchased AIS data from Marinetraffic.com for their development.

#### Landside model development

The traffic network of each case study was modeled, and emphasis was placed on the traffic network inside the port and the main transportation corridors that connected to its entrances and exits. The models represented accurate lane geometry, port configurations, and representative signal timings. These network attributes were based on publicly available satellite imagery, port visits, and other public sources. Vehicle demand and routing were estimated based on average annual daily traffic (AADT) values reported by the state departments of transportation. Actuated signal control was used for all signalized intersections using a representative timing plan. Additional network attributes were added in the model to achieve accuracy in the simulation. These attributes include vehicle speeds, conflict areas, reduced speed areas, etc.

#### Waterside model development

A Monte Carlo approach was taken to simulate vessel arrivals and dwell times. Using one year of historical data from each port, the proportion of vessels arriving in each hour of the day was calculated for major vessel types. Likewise, the number of hours each vessel type dwelled for was also calculated. Vessels were categorized into four broad groups: (1) containerized cargo, (2) non-containerized cargo, (3) tankers, and (4) passenger ships. These categories were chosen for their pervasiveness at each port and the unique loading and unloading characteristics of the cargo they carry. Table 1 provides a summary of vessel arrivals by cargo type at each of the study ports. The Monte Carlo simulation was then developed to replicate the arrival and dwell time characteristics of each vessel type. For example, the Monte Carlo simulation estimated the likelihood of a container vessel arriving between 7:00 and 8:00 a.m. and dwelling for 25 to 26 hours before departing. From this information, the Monte Carlo simulation generated random arrival and dwell times that corresponded to the observed probabilities.

**Table 1.** Number of vessel arrivals by cargo type between 01 January 2016 and 31 December 2016.

Ports	Container	Non-container	Tanker	Passenger
Miami	980	613	0	1055
Everglades	1908	474	338	778
Canaveral	134	231	205	2646
Charleston	3103	417	270	277
Jacksonville	2228	557	327	205
Palm Beach	819	156	0	215
Savannah	3943	149	336	1572

The data used to develop the Monte Carlo simulation contained 71,795 records of vessel arrivals, departures, and dwell times, from 1 January 2016 to 31 December 2016. Three additional months of data were also purchased for model validation. These data encompassed 1 January 2017 through to 31 March 2017. Vessel arrivals and dwell times were then simulated using the inverse transform sampling method. Fundamentally, this approach uses the inverse of the cumulative probability distribution function (CDF) to transform a uniformly distributed random value into an arrival time or dwell time that matches the historic distribution. Vessel arrivals were considered a discrete variable falling into any one of 24 time bins. Vessel dwell times, however, were a continuous variable, and unlike arrivals, which only have 24 possible bins, the number of 1-hour dwell time bins possible was nearly infinite. Therefore, when developing the dwell time distributions, the number of bins was limited to include most but not all observed dwell times. Furthermore, because it was desirable for the model to produce a continuous variable for the dwell time, the CDF of the dwell time was approximated to be a linear function. As a result, excessively long dwell times were truncated. However, this truncation was applied to less than 5% of the vessels. This could affect the model results, as dwell times were restricted to 95% of the possible dwell times observed. Moreover, the linear assumption was made out of convenience in the modeling approach, not because dwell times were observed to be conforming to a uniform distribution. This is a limitation of the current study.

#### Hybrid model integration

The Monte Carlo simulation only produced vessel arrivals and dwell times. VISSIM was used to simulate the physical infrastructure (vessels, channels, berths, etc.). In VISSIM, public transit vehicles were modified to represent the size and speed of the major vessel types within each port. Lanes were used to represent channels, and transit stops were used to represent berths. Vessel paths were drawn according to the geometry of the port and existing vessel traffic patterns. An average speed of 10 knots was used to accounts for transit speeds as well as a speed reduction during mooring and unmooring evolutions. The simulated vessel arrivals were modeled in VISSIM as public transport departure times. Vessel dwell times were modeled as occupants. The time required for each occupant to exit the public transit vehicle was set to 999 seconds. The simulated dwell times were converted to 999-second units and programed as occupants in VISSIM. Vessels in VISSIM were directed to the appropriate terminal, based on their cargo type. During port closures and times when a desired terminal was occupied, vessels queued outside of the port in a first-in-first-out fashion. Upon arriving at their desired terminal, vessels dwelled in accordance with the Monte Carlos simulation results.

### Scenario development

Three simulated scenarios were developed as part of this research. The first scenario represents a typical 30-day period of port operations beginning midnight of 29 September 2016 and ending at 11:59:59 on 28 October 2016. This scenario represented the *baseline case* and was titled as such: *Baseline case*. The *baseline case* scenario established a benchmark for port operations with which to compare subsequent results. The *baseline case* scenario results, like all of the modeled scenarios, constitute five simulated trials or “runs,” each having their own unique random number seed value. When conducting stochastic simulations, runs are needed to rule out the effects of randomness in the model that may influence the research findings. Seed values are used to vary the stochastic probabilities used between runs. While it would have been ideal to have additional simulation runs and seed values, each run required several days to complete. Furthermore, given the consistency between the five runs, significant improvements in model performance were not likely to result from additional runs.

The second scenario represents the impact of Hurricane Matthew and was titled *Hurricane Matthew*. Similar to the *baseline case* scenario, the simulation began on 29 September 2016 and had the same five runs and seed values as the *baseline case* (same arrival and dwell pattern). In this scenario, all port closures and re-openings observed during the actual Hurricane Matthew event were programmed into the models. The dates and times of the closures were provided by the Marine Safety Information Broadcast messages published by the Coast Guard. The ports of Miami, Everglades, and Canaveral closed on 5 October 2016 at 22:00 for 36, 38, and 82 hours, respectively. The port of Jacksonville closed on 6 October 2016 at 08:00 for 73 hours while the port of Savannah closed on 7 October 2016 at 08:00 for 120 hours. The port of Charleston closed for 61 hours from 16:00 on 7 October 2017.

In the simulation model, the port closures resulted in a queue of vessels waiting to enter the port. While no such queue existed during Hurricane Matthew (vessels did not wait just outside the port during the storm), the queue represented a backlog of vessels, which would have entered the port, if not for the storm and closure. This scenario also assumed that all vessels in a typical month would call their respective ports and not reroute due to the hurricane. Once each port was reopened in the simulation, the vessel backlog was serviced, resulting in increased demand placed on port infrastructure. Additionally, each vessel was required to wait until berthing space became available. It is noted that in the cases considered here, each port was assumed to be operating at 100% efficiency to service the queue of ships once it is opened. The methodology described in this paper could be adjusted to consider infrastructure damage, vessel rerouting, as well as a number of other possibilities not considered herein.

The third scenario represents the impact of additional investment in port capacity projects. The scenario models each port with additional berths. The amount of additional available berth space varied between ports and between vessel types. On average, an additional two to three vessels were serviced at the same time, at a single berth. The prior two scenarios treated berths as a single point of access, like a bus stop, whereas this scenario modeled the berths similar to a rail station, where multiple cars can be loaded and unloaded at the same time. The capacity was only limited by the port geometry and the size of the vessels occupying this space. In general, each berth could accommodate two vessels. However, several instances were observed where three vessels were accessing a single berth. This was possible because port geometry and vessel size varied.

Allowing additional “hypothetical” berthing space can represent a multitude of real-life scenarios that can greatly help port partners improve their resilience both on local and regional scales. The most obvious conclusion that can be drawn from this scenario was what infrastructure upgrades can affect the resilience of a port, whether it is additional berthing space or, where no more land exists to develop, additional equipment to expedite the onboarding and off boarding of cargo. This scenario can also represent the impact of increasing the labor to improve the efficiency of cargo operations. This scenario can also represent ship-to-ship transfers of cargo in order to reduce the vessel queue. For example, it can be used to assess the impact of tanker vessels conducting lightering operations at a tanker barge. These results can assist port partners with pre-storm planning, more specifically potentially pre-staging tanker barges to expedite the clearing of the tanker vessel queue. Multiple results from a scenario of this type can be considered, in support of increasing the resilience of the ports considered. This scenario was titled *Capacity Enhanced* in the results.

## Model calibration and validation results

Four separate data sets were used in the calibration and validation of the simulation model. The first was a 12-month data set acquired to generate the simulation models. This data set was referred to as the *12-Month Cal* (calibration) data set. Next, the simulation model was used to generate 10, one-year periods. This allowed the model to generate enough observations to verify the accuracy of simulation predictions. This data set is titled *10 Year Sim* (simulation). Next, five 30-day simulation runs (with five unique seed values) were developed and evaluated for their accuracy. This data set is titled *30 Day Sim* (simulation). Finally, the validation data set purchased from MarineTraffic.com was also shown to validate the model findings. The validation data set is titled *3 Month Val* (validation).

**Figure 2.**
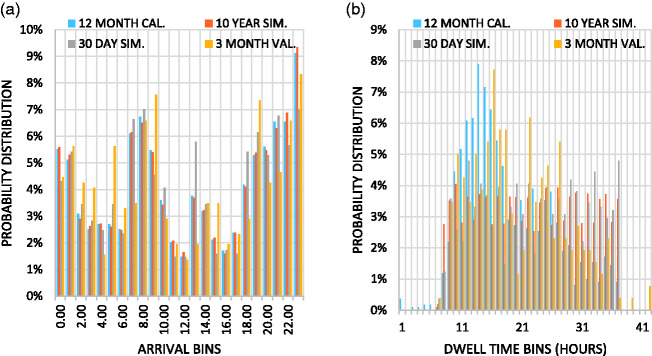
(a) Port of Jacksonville container vessel arrival calibration and validation results. (b) Port of Jacksonville container vessel dwell time calibration and validation results.

The model calibration and validation are conducted in two stages. First, a qualitative assessment was investigated to provide a consensus of how the models performed. This was accomplished by plotting the historical probability distribution functions (*12 Month Cal*. and *3 Month Val*.) alongside the distribution functions generated by the simulation model (*10-Year Sim* and *30-Day Sim*). Figure 2(a) shows the arrival distribution functions (historic and simulated) for container vessels at the Port of Jacksonville. The figure suggests an overall consistency between the four data sources. In general, distribution plots with a higher number of observations and more consistency within the 12-month historical distributions show more consistent model and validation results. Likewise, distributions built upon fewer observations or ones with inconsistent observations made during the 12-month historical data set, produced less stable model results. Overall, the plotted distributions represented the essence of the operations at each port. Figure 2(b) shows the dwell time probability distribution functions for the Port of Jacksonville. Figure 2 suggests that dwell times tended to under preform. This was expected because of the linearity assumption made in the modeling process. Furthermore, predicting which hour a vessel would arrive was fundamentally easier than predicting how many hours the vessel is likely to dwell.

The second calibration and validation approach uses the coefficient of determination, also known as the *R-Squared* value, to quantitatively evaluate the model’s goodness-of-fit. For the purpose of this study, the calibration results were estimated by the coefficient of determination when comparing the 12 Month Cal data set to both the 10-year simulation and the 30-day simulation. Table 2 provides the calibration results for vessel arrival and dwell times.

**Table 2.** Vessel arrival and dwell time calibration results.

Port	Container		Non-container		Tanker		Passenger	
	10 Yr	30 Day	10 Yr	30 Day	10 Yr	30 Day	10 Yr	30 Day
Miami	0.998	0.704	0.994	0.706	N/A	N/A	0.999	0.078
Everglades	0.986	0.485	0.97	0.476	0.974	0.422	0.997	0.947
Canaveral	0.94	0.376	0.955	0.54	0.929	0.381	0.999	0.988
Jacksonville	0.995	0.821	0.941	0.581	0.951	0.302	0.999	0.936
Savannah	0.997	0.824	0.985	0.754	0.942	0.478	0.999	0.981
Charleston	0.998	0.916	0.96	0.358	0.857	0.176	0.995	0.828
Vessel dwell time calibration results								
Miami	0.002	0.001	0.042	0.007	N/A	N/A	0.37	0.212
Everglades	0.271	0.114	0.17	0.221	0.499	0.31	0.248	0.184
Canaveral	0.106	0.018	0.017	0.002	0.158	0.154	0.469	0.516
Jacksonville	0.441	0.341	0.308	0.069	0.404	0.132	0.181	0.595
Savannah	0.314	0.35	0.025	0.013	0.003	0.008	0.099	0.104
Charleston	0.37	0.473	0.012	0.063	0.15	0.033	0.287	0.249

The coefficients of regression suggest accurate results with regard to when vessels entered the port. *R-squared* values ranged between a low of 0.302 and a high of 0.999, with the majority of the results above 0.90. The dwell time calibration results show a range of *R-squared* values between 0.001 and 0.516. This suggests that the model struggled to reproduce the exact dwell times seen in the *12 Month Cal* data set. This was expected due to the simplified modeling approach taken for dwell times and the assumption of a linear CDF.

Validation was measured by the *R-squared* value generated by the comparison of the simulation data sets and the *3 Month Val* data set. Table 3 provides the validation results. In general, the *R-squared* values found during validation were significantly lower when compared to calibration. The *R-squared* values for vessel arrivals ranged between 0.001 and 0.729. One reason for the lower *R-squared* values could be that the validation data set was taken over a consecutive three-month period. The simulation models developed did not account for seasonable variability, and therefore, seasonal changes in cargo shipments were not reflected in the model.

**Table 3.** Vessel arrival and dwell time validation results.

Port	Container		Non-container		Tanker		Passenger	
	10 Yr	30 Day	10 Yr	30 Day	10 Yr	30 Day	10 Yr	30 Day
Miami	0.021	0.001	0.354	0.372	N/A	N/A	0.421	0.007
Everglades	0.355	0.045	0.036	0.021	0.331	0.142	0.639	0.593
Canaveral	0.001	0.005	0.072	0.007	0.044	0.037	0.556	0.571
Jacksonville	0.584	0.413	0.024	0.105	0.095	0.113	0.317	0.405
Savannah	0.729	0.624	0.041	0.050	0.005	0.004	0.426	0.451
Charleston	0.662	0.630	0.028	0.168	0.007	0.148	0.510	0.394
Vessel dwell time validation results								
Miami	Null	Null	0.014	0.003	N/A	N/A	0.034	0.174
Everglades	0.173	0.027	0.093	0.041	0.392	0.259	0.240	0.153
Canaveral	0.054	0.009	0.001	0.001	0.033	0.010	0.420	0.472
Jacksonville	0.383	0.325	0.018	0.001	0.073	0.082	0.003	0.001
Savannah	0.300	0.255	0.001	0.001	0.001	0.001	0.086	0.066
Charleston	0.321	0.387	0.003	0.001	0.028	0.004	0.215	0.184

Overall, the models were generally successful in reproducing the arrival patterns of the 12-month calibration data set. However, significant deficiencies were observed in replicating vessel dwell times. This was because the model assumed a linear cumulative distribution and by extension, uniform distribution of vessel dwell times. While this assumption significantly affected the coefficient of regression, it still provided dwell time values that were generally agreeable with the observed values. This was evident in a review of the dwell time distribution plots, an example of which was provided in Figure 2(b). The model also performed poorly with regard to validation. Arrival patterns and dwell times were both shown to have low coefficients of regression values. This was likely because noteworthy discrepancies existed between the arrival and dwell time patterns seen between the 3-month validation data set and the 12-month calibration data set. These discrepancies were likely caused by seasonal fluctuations in vessel traffic and changes in cargo demand between years. Ultimately, the decision was made to move forward with the models for two reasons: (1) the models showed general agreement with the observations and (2) the models were used as a tool to measure the difference between simulated scenarios. Any errors seen in one model were replicated in the next. By only investigating the difference between the model results, assumptions made in the modeling process were not likely to significantly affect the overall findings of the work.

## Scenario results

**Figure 3.**
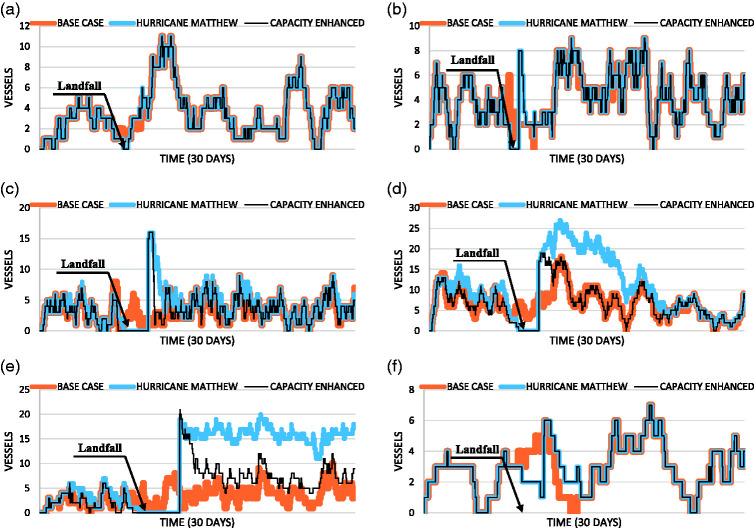
(a) Port of Miami container cargo vessel occupancy. (b) Port Everglades container cargo vessel occupancy. (c) Port Canaveral passenger vessel occupancy. (d) Port of Jacksonville container cargo vessel occupancy. (e) Port of Savannah container cargo vessel occupancy. (f) Port of Charleston container cargo vessel occupancy.

The simulation results from VISSIM provide a wide range of output parameters. The key measure of effectiveness (MOE) selected to present here was port occupancy. Port occupancy was defined for this research as the number of vessels of a particular cargo type occupying the port at a given moment in time. In this sense, occupancy provides an indication of port capacity. As a port becomes congested after the closure, capacity and by extension occupancy, become a significant concern for port managers. In general, the most frequent cargo type observed at the case study ports was containerized cargo. This was true for all but one port: Port Canaveral, which typically saw the highest traffic from passenger vessels. Because the analysis of port capacity was the primary consideration, only the cargo types with the highest demand at each port were described here. A preliminary analysis of the lower demand cargo types found little impact to port operations caused by the storm closures. Figure 3 shows occupancy for the highest demand cargo type at each port. The orange line represents the *baseline case* scenario over the 30-day event timeline in the absence of the disruption. The blue line shows the modeled port operations during Hurricane Matthew, and the black line represents the scenario involving enhanced port capacity. These figures represent time-dependent resilience plots for port occupancy for a decreasing service system, similar to Figure 1(b).

Figure 3(a) and 3(b) shows the container vessel occupancy for the Port of Miami and Port Everglades, respectively. In general, the impact of the closure on containerized cargo vessels at these ports was minor. This was expected because these ports were located furthest from the storm’s path and experienced relatively short closures of just 36 and 38 hours, respectively. Table 4 provides the results from the resilience analysis (equation (4)) at each port. The ports of Miami and Everglades showed a strong ability to adapt and recover from the disruption, as evident by high scores in the Disruption and Recovery stages of the event. Both ports received lower marks during the Absorption state. This too was expected, as a closure results in a vessel evacuation and a drastic drop in port occupancy. The results also suggest there was no apparent impact on the capacity enhancements at either port. Again, this was likely because these ports were out of the storm’s direct path and only experienced a minimal disruption, i.e., capacity enhancements were not needed to address the short queues.

**Table 4.** Resilience analysis results.

Port	State	*Hurricane Matthew*	*Capacity enhanced*
Miami	Absorption	0.356	0.356
	Disruption	0.826	0.826
	Recovery	0.851	0.851
	**Resilience**	**0.250**	**0.250**
Everglades	Absorption	0.193	0.193
	Disruption	0.556	0.556
	Recovery	0.694	0.694
	**Resilience**	**0.074**	**0.074**
Canaveral	Absorption	0.114	0.066
	Disruption	0.533	0.316
	Recovery	0.691	0.861
	**Resilience**	**0.042**	**0.018**
Jacksonville	Absorption	0.146	0.146
	Disruption	0.878	0.600
	Recovery	0.204	0.923
	**Resilience**	**0.026**	**0.081**
Savannah	Absorption	0.268	0.131
	Disruption	0.000	0.744
	Recovery	0.000	0.260
	**Resilience**	**0.000**	**0.025**
Charleston	Absorption	0.525	0.525
	Disruption	0.970	0.970
	Recovery	0.264	0.264
	**Resilience**	**0.135**	**0.135**

Figure 3(c) shows the passenger vessel occupancy results for Port Canaveral. The figure suggests a significantly larger backlog of vessels following the reopening of the port, as compared to the ports of Miami and the Everglades. While it is unlikely that passenger vessels would queue in such a way, the analysis does provide for a theoretical examination of the passenger vessel capacity at the port. The resilience analysis results, provided in Table 4, suggest the port struggled to absorb the impact of the closure. However, similar to the ports further south, Canaveral experienced a mild Disruptive and Recovery state, suggesting the port facility was able to adapt and recover efficiently to a hurricane event. The modeled capacity enhancements resulted in a lower resilience rating of the port. This was evident in lower Absorption and Disrupted state values. Because the capacity enhancements allowed the port to more quickly evacuate following the closure, the fall in vessel occupancy occurred more drastically when compared to the *Hurricane Matthew* scenario. This, in turn, initiated the Disrupted state earlier. Because the port was modeled to reopen at the same time, regardless of when the last vessel evacuated, the Absorption state value was adversely impacted by the rapid clearance of vessels from the port. However, the capacity enhancements significantly reduced the time required to service the queue and provided a much stronger recovery value than the *Hurricane Matthew* scenario. Overall, the enhancements provided a 23-hour time-saving.

Figure 3(d) shows the containerized cargo vessel occupancy at the Port of Jacksonville. The figure clearly shows a significant impact from the closure and an extended duration of time needed to address the backlog of cargo vessels. The port did not return to a Stable Post-Recovery state until 21 21 October 2016 at 9:00 a.m., over two weeks following the closure. Similar to the other ports, the Port of Jacksonville struggled to absorb the impact of the closure. In both the *Hurricane Matthew* and *Capacity Enhanced* scenarios, the Disrupted State represented the same duration of time. However, the *Hurricane Matthew* scenario resulted in a significant longer overall period of poor port performance. This led to the Disrupted state representing a shorter relative duration, which increased the resilience rating of the Disrupted state. Because the *Capacity Enhanced* scenario was able to reduce the duration of the event, the fixed Disrupted state period represented a higher proportion of the event period, and thus, a lower resilience valuation of the Disrupted state for this scenario. The *Capacity Enhanced* scenario suggested a possible saving of approximately 251 hours or nearly 10½ days.

Figure 3(e) shows the containerized cargo vessel occupancy at the Port of Savannah. The figure again shows a significant impact of the closure on the port as well as extended duration of time needed to address the backlog of cargo vessels. The *Hurricane Matthew* scenario did not return to a Stable Post-Recovered state by the end of the simulation period, three weeks after the initial closure. The *Capacity Enhanced* scenario, however, was able to address the queue adequately in 83 hours. The discrepancy between the two recoveries was likely because the Port of Savannah typically operates at or near capacity. With little excess capacity during routine conditions, the simulation suggests the port would likely struggle to recover from a disruptive event. Because the port did not fully recover from the *Hurricane Matthew* event, it was effectively not resilient (zero resilience valuation). However, if the simulation period was to be extended, it is likely the port would ultimately return to a Stable Post-Recovered state.

Figure 3(f) shows the containerized cargo vessel occupancy at the Port of Charleston. The figure shows a relatively mild impact of the closure on the port. The port returned to a Stable Post-Recovered state 61 hours after closing. The Port of Charleston demonstrated the highest resilience of any of the study ports with regard to absorption and adaptability. Investigating the port occupancy observed in the two weeks prior to landfall, the figure suggests that the Port of Charleston experiences periodic fluctuations in vessel arrivals. This would suggest the port experienced cyclical periods of high demand, followed by periods of low demand. Therefore, the port was likely designed with a relatively high capacity compared to its average daily vessel transits. Consequently, the port was able to use the excess capacity to service the vessel queue and increase the absorption and recovery resilience valuations. Because the port already had excess capacity, the *Capacity Enhanced* scenario did not significantly affect port occupancy or the resilience estimations.

The impact of Hurricane Matthew to the region was significant. While most of the effects were concentrated in the northern ports, measurable losses were observed at the Ports of Miami and Everglades. In general, the resilience results suggest the ports of Jacksonville and Canaveral were the most impacted. This makes sense given the storm’s path. Interestingly, the port of Charleston fared better after the storm than most other ports in the region. Given the peak and wane nature of container arrivals at the port of Charleston, it is likely that the port was better suited to accommodate the surge in demand after the port was reopened. Overall, the simulated results indicate what might have occurred, had the U.S. Coast Guard, U.S. ACE, and port managers not taken actions during Hurricane Matthew. The results suggest the adverse effects of this major disruptive event would have likely been significantly more disastrous. However, the planning and recovery actions pursued, in reality, mitigated these impacts. The analysis and results suggest an additional opportunity for enhanced regional resilience. While many of the northern ports experienced significant queues and delays, the southern ports were operating at normal capacity within a few days of reopening. Therefore, for cargo that can be transported over the road network, it makes sense to reroute these vessels to neighboring ports. This would decrease the initial queue or backlog of vessels needing to be serviced by the most significantly impacted ports. Decreasing the initial queue would likely have an exponential impact on vessel-hours lost because the relationship between initial queue and recovery time was observed to be nonlinear. This was also seen for the modeling of the enhanced capacity scenarios. For example, at the Port of Jacksonville, the capacity enhancements allowed the port to accommodate the sudden surge in vessel traffic, leading to the port returning to Stable Post-Recovered state sooner. This mitigated the cascading impact of new vessel arrivals joining the queue, seen in the *Hurricane Matthew* scenario.

## Conclusion

This paper presented two novel frameworks for assessing port resilience. First, the paper documents the development of a new approach to estimating system resilience, applicable to a wide range of systems and events. Second, the paper describes a hybrid multimodal approach to simulating port operations. The developed methodology was applied to six ports along the southeast US coastline. In addition, while the methodology was used to develop a retrospective of the 2016 Hurricane Matthew disruption, the proposed approach can easily be used for planning purposes. For example, the *Capacity Enhanced* scenario was developed to assess the impact of the disruption had additional capacity had been developed at these ports. The modeling approach can serve as a tool for assessing and planning for the resilience of a single port or several ports in region in response to a variety of disruptive events. The tool provides opportunities for exploring the consequences of alternative decisions in responding to disruptions.

In general, the results of the research show the benefits of the simulations in quantifying the impact of an event and how the information gained from such an analysis can be beneficial when evaluating alternatives. In the case studies considered, the impact of enhanced service capacities at ports in clearing backlogs was shown. Such quantitative assessments provide meaning, context, and relevance to port stakeholders that may not be readily apparent at face value. This research also showed that AIS data could be utilized to create new methods and metrics for the assessment of resilience in maritime systems. This methodology advances the field of disaster science by expanding on the concepts first proposed by Henry and Ramirez-Marquez (2012) and Baroud et al. (2014). The time-dependent performance models showed the cascading effects of disruptions and quantified the benefits gained by recovery efforts in a time-progressive series. The data showed, in quantifiable terms, reductions in performance resulting from the simulated disruption. On a broad level, these findings also represent some of the first steps toward the development of standardized metrics for quantifying maritime transportation system resilience.

Future work could include refining the proposed resilience equations as well as developing a systematic approach to identifying the points of interest from the time-dependent resilience plots. Currently, these values were selected through an analysis of the data and best judgement. A systematic approach could be developed to refine this and eliminate subjectivity. Furthermore, weights could be applied to the stages of the disruption to provide a more contextualized assessment. This research also adopted the National Academies definition of resilience, which calls for a system to absorb, adapt, and recover. This led to the development of equation (4) and ultimately led to low resilience values. This could be improved upon in future work by combining the performance during the various stages in a different way. Additional work could also improve upon the Monte Carlo simulation by eliminating some of the assumptions used in this paper. Furthermore, a model that connects each of the study ports over both land and sea could demonstrate the resilience implications of rerouting and vessels and freight vehicles. In addition, the incorporation of rail traffic would also benefit the model.
